# β-Lactoglobulin and Glycodelin: Two Sides of the Same Coin?

**DOI:** 10.3389/fphys.2021.678080

**Published:** 2021-05-20

**Authors:** Lindsay Sawyer

**Affiliations:** School of Biological Sciences, IQB3, The University of Edinburgh, Edinburgh, United Kingdom

**Keywords:** β-lactoglobulin, glycodelin, *PAEP*, biological function, lipocalin

## Abstract

The two lipocalins, β-lactoglobulin (βLg) and glycodelin (Gd), are possibly the most closely related members of the large and widely distributed lipocalin family, yet their functions appear to be substantially different. Indeed, the function of β-lactoglobulin, a major component of ruminant milk, is still unclear although neonatal nutrition is clearly important. On the other hand, glycodelin has several specific functions in reproduction conferred through distinct, tissue specific glycosylation of the polypeptide backbone. It is also associated with some cancer outcomes. The glycodelin gene, *PAEP*, reflecting one of its names, progestagen-associated endometrial protein, is expressed in many though not all primates, but the name has now also been adopted for the β-lactoglobulin gene (HGNC, www.genenames.org). After a general overview of the two proteins in the context of the lipocalin family, this review considers the properties of each in the light of their physiological functional significance, supplementing earlier reviews to include studies from the past decade. While the biological function of glycodelin is reasonably well defined, that of β-lactoglobulin remains elusive.

## Introduction

When β-lactoglobulin (βLg) was first isolated by Palmer, 1934 there can be little doubt that nobody realized that the protein would remain something of a puzzle 85 years later. βLg, a significant component of cow’s milk, is a member of the ancient and widespread protein family that came to be named the lipocalins (Pervaiz and Brew, 1985). The protein is abundant and easily prepared so that it has served as a convenient test-bed for essentially every molecular technique from absorption spectroscopy (Townend et al., 1960) to X-ray crystallography (Crowfoot and Riley, 1938) and zeta-potential measurement (Chen and Dickinson, 1995) and pretty much everything else in between. Its ready availability has also led to redox (Conway et al., 2013; Corrochano et al., 2018), enzymic (Li et al., 1990; Gowda et al., 2017, 2018) and co-factor (Pérez et al., 1992) properties being ascribed though that is hardly surprising. What is not always clear, is whether any of these observations have any direct relevance to the physiological function.

The report by Futterman and Heller (1972) that βLg bound retinol was almost certainly unexpected as the focus of the paper was retinol binding to retinol-binding protein (RBP) and a convenient “blank” was required. The correct sequence of βLg was published the same year (Braunitzer et al., 1972), and the sequence similarity between βLg and α2u-microglobulin was noted by Unterman et al., 1981. However, it was not until the publication of the sequence (Rask et al., 1979) and structure (Newcomer et al., 1984) of retinol-binding protein that it became clear there was a close structural relationship (Godovac-Zimmermann et al., 1985; Sawyer et al., 1985). Since then all biological kingdoms have been found to contain family members, the lipocalins (Pervaiz and Brew, 1985), the functions of the majority being associated with communication in its broadest sense: transport (serum retinol binding protein), camouflage (insecticyanin and crustacyanin), stress response (apolipoprotein D and α_1_-acid glycoprotein), marking (mouse urinary protein and darcin), and some have enzymic activity (prostaglandin-D synthase, reductase, and plant epoxidase) (Flower, 1996; Åkerström et al., 2006). To date there are some 1,500 entries under “lipocalin” in the Protein Data Bank (Berman et al., 2003) and more than 46,000 in the UniProtKB database (The UniProt Consortium, 2019). However, this review will concentrate on a tiny subset of the family, βLg and glycodelin (Gd, PAEP) described by Gutierrez et al. (2000) and Sanchez et al. (2006) as Clade IV of a general lipocalin classification, paying particular attention to their physiological function (Pérez and Calvo, 1995).

**FIGURE 1:**
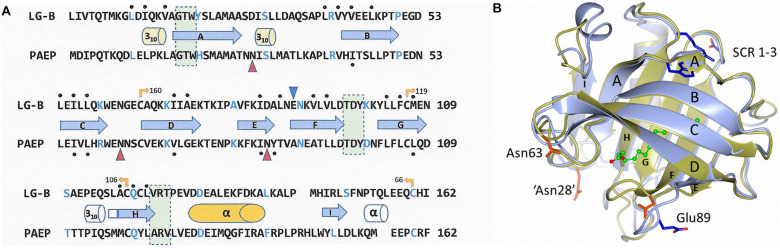
**(A)** Alignment of the protein sequence of cow β-lactoglobulin B variant (LG-B) with that of human glycodelin (PAEP, Gd) showing the structurally conserved regions (SCR) in the lipocalin family boxed in pale green. Every 10th amino acid is colored blue and the secondary structural elements shown as cylinders for helices and arrows for β-strands, labeled as they are in the crystal structure. Uncolored structural elements appear in only one structure both structures and disulphide bridges are shown as yellow arrows with the partner residue number. Glycosylation sites in Gd are marked with red triangles, that at 85 being unused, while the ligand-gating Glu89 in βLg is shown as a blue triangle. The residues lining the central calyx are marked above/below with black dots, from which it will be seen that the cavity in Gd is very much smaller than that in βLg (Schiefner et al., 2015). **(B)** A cartoon showing the remarkable similarity of monomers of βLg (PDB: 1gxa, blue-gray) and Gd (PDB: 4r0b, olive). The βLg structure has a molecule of palmitate bound within the central calyx (carbon atoms in green, oxygen in red). The β-strands are labeled as in **(A)**. The helix is at the rear and the structurally conserved regions (SCR1-3) are indicated by residues Trp19, Asp98 and Arg124 in blue, all on the outer surface at the foot of the calyx. Glu89 in βLg which is on the EF-loop in the open position is close to the unglycosylated Asn85 in Gd shown in pink. The other residues in pink, Asn63 and Glu28, show the positions of the glycosylation present in native Gd. The mutation Asn28Glu was necessary to improve the solubility of the cloned Gd used in the X-ray analysis. Dimerization involves the I-strand in both proteins but there is significantly greater interaction in Gd (1170 Å^2^ buried surface area) compared to βLg (530 Å^2^) despite the remarkable overall similarity (the rmsd of the 160 Cα atoms is about 0.65Å). The carbon chain of palmitate is green. Figure drawn by CCP4mg (McNicholas et al., 2011).

Throughout the 1980s as comparison techniques became more robust, 3-dimensional molecular structures began to be used to infer homology as a complement to sequence-based techniques (Figure 1). This was particularly important when the pairwise identity of sequences dropped below 25–30% (Rost, 1999). βLg/RBP and βLg/α2u-microglobulin sequence comparisons (pairwise sequence identity of approximately 25%) highlighted their potential evolutionary relationships (Pervaiz and Brew, 1985). Other proteins, however, show even lower pairwise sequence identities and yet their membership of the same homology family became apparent when the folds revealed by the tertiary structure were found to be similar. The lipocalin fold is an 8-stranded up-down β-barrel open at one end forming what has come to be known as the calyx, with a 3-turn α-helix packing on the outer surface and usually a ninth β-strand located under the helix also on the barrel surface. As more members of the family emerged, three structurally conserved regions (SCR, Figure 1) were identified that served as signatures (formally, synapomorphies) and which are present in the vast majority of lipocalins: almost all possess SCR1 and SCR3, and many also contain SCR2. Intriguingly, all three of the SCRs are found on the solvent side of the foot of the calyx, implying some similarity of function (North, 1989). In addition, in many lipocalins including, Gd and βLg, there are conserved, intramolecular disulphide bridges. Now that DNA sequencing of whole genomes can be readily achieved, it is found that within the lipocalin family the intron/exon boundaries are also well conserved despite poor sequence identity (Salier, 2000; Sanchez et al., 2006).

**FIGURE 2:**
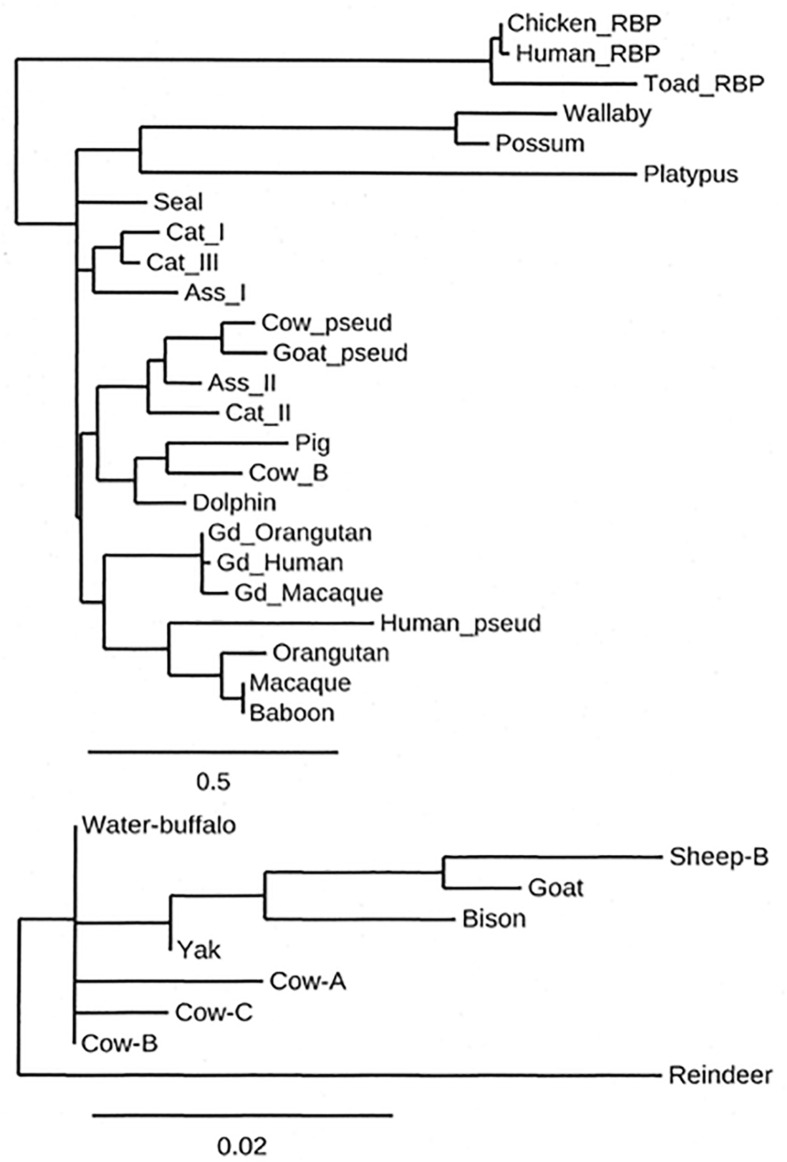
A cladogram showing the relative amino acid sequence relationship of a selection of β-lactoglobulin (βLg), glycodelin (Gd) and retinol-binding protein (RBP) sequences, RBP being included as an outgroup to the βLgs and Gds. The inset shows the relationship between domesticated ruminant β-lactoglobulins. Note the difference in the branch-length scales. The amino acid sequences were aligned by CLUSTAL-Ω (Sievers et al., 2011) using its default parameters, and the cladogram was produced by the program PHYLOGENY.fr (Dereeper et al., 2008). The sequences used were UniProtKB (The UniProt Consortium, 2019) unless otherwise mentioned. *Lactoglobulins*: Platypus (F6SX48), Wallaby (Q29614), Possum (Q29146), Orangutan (NCBI: XP_002820419), Macaque (NCBI: XP_005580520), Baboon (O77511), Pig (P04119), Cow B variant (P02754), Beluga (NCBI: XP_022433973.1), Dolphin (A0A6J3RLE3), Cat I (P33687), Donkey I (P13613), Donkey II (P19647), Cat II (P21664), Cat_III (P33688), Fur Seal (A0A3Q7NUB2-1), Reindeer (Q00P86), Yak (L8J1Z0), Bison (NCBI: XP_010855058), Sheep B (P02757), Water buffalo (P02755), and Goat (P02756). *Glycodelins*: Gd_Human (P09466), Gd_Orangutan (NCBI: XP_00377753), Gd_Macaque (Q5BM07), *Pseudogenes*: Cow βLg pseudogene (Genbank: Z36937), Goat βLg pseudogene (Genbank: Z47079), Human βLg pseudogene (Ensembl: hg38_dna.[2298:6285].sp.tr), *Retinol Binding Proteins*: Toad RBP (P06172), Human RBP (P02753), and Chicken RBP (P41263).

Because members of the lipocalin family are found in all kingdoms, the ancestral lipocalin must have appeared long before the amniotes emerged around 250 million years ago (Mya) and it appears likely that there was already a form of animal skin secretion which was to develop into what is now referred to as lactation (Oftedal, 2002, 2013; Newman et al., 2018; Sharp et al., 2020). These skin secretions could provide sustenance and protection from infection for offspring and this process is still found in the egg-laying monotremes, animals whose young are produced in an immature form and require both feeding and protection. As the offspring matures, the composition of the secretion changes (Tyndale-Biscoe and Janssens, 1988; Lefèvre et al., 2010; Kuruppath et al., 2012; Sharp et al., 2020). The development of secretory cells associated with a specific organ, the teat, appears to have occurred around 165 Mya subsequently followed by the development of the true placenta and the emergence around 148 Mya of the eutheria or placentalia (Oftedal, 2002, 2013; Lefèvre et al., 2010). Many of the proteins that are present in the milk of today’s placental species were present 160 Mya (Oftedal, 2013; Vilotte et al., 2013). The origin of βLg therefore must have been at least 160 Mya based on the secretions provided by the ancestral monotremes for their offspring (Oftedal, 2000; Joss et al., 2009; Lemay et al., 2009; Skibiel et al., 2013; Sharp et al., 2020). Gd, the other protein in Clade IV (Salier, 2000; Sanchez et al., 2006) is more recent as has been pointed out by Oftedal (2013). Figure 2 shows a simple representation of the closer relationship of the lactoglobulins and the glycodelins than of the rest of the lipocalins, represented by RBP. It is the lineage leading to the placentalia upon which this review focuses.

Speculation that Gd might be the precursor of βLg (Kontopidis et al., 2004; Cavaggioni et al., 2006; Sawyer, 2013) is wrong, a mumpsimus, for several reasons. Lactation preceded placentation and, as βLg is found in monotremes as well as marsupials and eutheria, it was well-established before the emergence, no longer than 60 Mya, of the endometrial protein Gd (Oftedal, 2002; Lefèvre et al., 2010; Schiefner et al., 2015). Gd is glycosylated (Julkunen et al., 1988; Dell et al., 1995) and most, if not all, of its distinct functions depend upon this glycosylation (Halttunen et al., 2000; Seppälä et al., 2007; Lee et al., 2016) so that it is improbable that a post-translationally modified form could have arisen before the polypeptide itself! Further, Gd has no (Koistinen et al., 1999) or at least a significantly lower (Breustedt et al., 2006; Schiefner et al., 2015) affinity for hydrophobic ligands. Mutations Asp28Asn and Glu65Ser in βLg which form the glycosylation sites in Gd could have occurred earlier but there is little evidence, although glycosylation of βLg has been reported. There appears to have been a rare genetic mutation discovered only in the individual analysis of a large number of Droughtmaster animals (Bell et al., 1970, 1981) or possibly more commonly, in the milk of the domestic pig, *Sus scrofa* (Hall, 2010). However, in this latter case the glycosylation is O-linked through Thr4, unlike the N-linking in Gd (Dell et al., 1995), and in βLg-Dr (Bell et al., 1981). Another vertebrate lipocalin, lipocalin-2 (LCN2) or neutrophil gelatinase-associated lipocalin is N-glycosylated but at a site on the C strand, distinct from those in Gd (Holmes et al., 2005; Bandaranayake et al., 2011).

Lactation, a characteristic of mammals, produces a fluid rich in protein, fat and sugars, the exact proportions of which vary considerably across species and through lactation (see for example Table 1 and Jenness and Sloan, 1970; Martin et al., 2013; Powers and Shulkin, 2016; Goulding et al., 2020). βLg is widely but not universally distributed – it is absent from the milk of rodents and lagomorphs (glires), camels, and humans although a pseudogene, *PAEPP1* (Hunt et al., 2018), is present but not expressed, close to *PAEP* on human chromosome 9. Some species, for example horses and cats, express paralogs and cows and goats have a pseudogene, related to one of these paralogs (Passey and Mackinlay, 1995; Folch et al., 1996). Most work on Gd has been on primates, especially humans and Schiefner et al. (2015) report its presence only in the old and new world monkeys and the hominids. However, there are isolated reports of its occurrence elsewhere. For example, in a proteomic study of dairy herd fertility (Koh et al., 2018), the plasma exosomes of heifers of low fertility contain the sequence of Uniprot G5E5H7 reported to be that of the gene *PAEP*, Gd. It is in fact the sequence of βLg-B. *In situ* hybridization on rat genital tract and PCR followed by sequencing has identified 100 bases of mRNA sharing “100% homology” with human glycodelin (Keil et al., 1999) and polyclonal antibodies to human Gd cross-react with rat reproductive and lung tissues (Kunert-Keil et al., 2005, 2009; Erdil et al., 2020). While false positive results arising in antibody cross reactivity experiments are not uncommon, the apparent presence of Gd in the rat by mRNA hybridization (Keil et al., 1999) remains something of a mystery. Putative *PAEP* pseudogenes have been identified in the genomes of tarsier, rat, rabbit and dolphin (Moros-Nicolás et al., 2018). Rodents and lagomorphs do not express βLg, though dolphin (Pervaiz and Brew, 1986) and tarsier (Schiefner et al., 2015) do. Lemay et al. (2009) report that there has been a loss of a section of DNA coding for amongst others, βLg, in glires since they were unable to find it and an evolutionary break point exists in the same region between the rodent and human genomes (Murphy et al., 2005). This reasoning would explain the absence of the milk protein in glires.

**TABLE 1:** Milk composition for a range of animals^1^.

Source		Fat %^2^	Protein %^2^	Whey %^2^	βLg^2^ mg/ml	PAEP^3^	References
*Bos taurus*	Cow	3.7	3.6	0.6	3.0	?	
*Capra hircus*	Goat	4.5	2.9	0.4	1.4		
*Ovis aries*	Sheep	7.4	5.5	0.9	2.8		
*Cervus elaphus L.*	Red deer	19.7	10.6		2.9		
*Sus scrofa domesticus*	Pig	6.8	4.8	2.0	0.6^2^		
*Canis familiaris*	Dog	12.9	7.9	2.1	10.1		
*Felis catus*	Cat	4.8	7.0	3.3			
*Equus caballus*	Horse	1.9	2.5	1.3	2.6		Wodas et al. (2020)
*Tursiops truncatus*	Dolphin	33.0	6.8	2.9	16.2		
*Callorhinus ursinus*	Fur seal	53.2	9.6	4.9	25^4^		Ashworth et al. (1966); Sharp et al. (2020)
*Macropus rufus*	Red kangaroo	3.4	4.6	2.3			
*Macropus eugenii*	Tammar wallaby		3.6	2.6	6.7^5^		Green and Renfree (1982), Lefèvre et al. (2007)
*Didelphis marsupialis*	Opossum	7.0	4.8	2.0			
*Tachyglossus aculeatus*	Echidna	9.6	12.5	5.2	15^6^		
*Papio cynocephalus*	Yellow baboon	5.0	1.6	0.5		Yes	Buss (1978)
*Macaca mulatta*	Rhesus monkey	4.0	1.6	0.5	2.8	Yes	Kunz and Lönnerdal (1994)
*Homo sapiens*	Human	3.8	1.0	0.6	∼0^7^	Yes	
*Mus musculus*	Mouse	13.1	9.0	2.0	0.0		
*Rattus norvegicus*	Rat	10.3	8.4	2.0	0.0	?	
*Oryctolagus cuniculus*	Rabbit	12.9	12.3	3.7	0.0		Maertens et al. (2006)

*^1^These data are mostly taken from Jenness and Sloan (1970) in which protein, in most cases, is listed as casein and whey protein. Here “protein” is given as the sum of the two. Figures are indicative as amounts vary within species and throughout lactation. βLg values are mostly from Sawyer (2003) and references therein. ^2^Protein is expressed as a percentage with no distinction made between g/100 g or g/100 mL, the specific gravity of milk being close to 1 g/mL. ^3^PAEP refers to whether the presence of glycodelin has been verified. A question mark means that it has been reported. A blank means there is no information. ^4^The βLg concentration is calculated based upon the percentage of nitrogen, relative to the total nitrogen in Ashworth et al. (1966). ^5^Estimated from the total protein and the relative abundance of expressed sequence tags. ^6^Estimated from the relative abundance of the expressed sequence tags and the crude skim milk protein concentration. ^7^An unexpressed coding sequence for human βLg has been identified (PAEPP1) but reports of its presence in milk are based upon immunological cross reactivity and it is known that lactoferrin can cross-react and ingested cow’s milk peptides have been found in human milk.*

Since *PAEP* is now also used to describe the βLg gene, rather than *LGB* or *BLG* (e.g., Elsik et al., 2016; Hunt et al., 2018; HGNC, 2020), it has become difficult to be certain as to which protein is present without protein analysis. Schiefner et al. (2015) use the presence of the glycosylation sites at 28 and 63 to distinguish Gd from βLg which is a convenient method though it does not necessarily confirm expression. The reports by Azuma and Yamauchi (1991) and Kunz and Lönnerdal (1994) of a βLg-like protein with an Mr > 20,000 from the milk of the Rhesus monkey, *Macaca mulatta*, predate the comparative proteomic analysis of Beck et al. (2015) which describes this protein as Gd. However, the short N-terminal sequence (Azuma and Yamauchi, 1991) matches that of βLg in Schiefner et al. (2015), described in the UniProt database as, *inter alia*, ‘Lipocln_cytosolic_FA-bd_dom domain-containing protein’ and the two functional glycosylation sites are absent.

In summary, the ancestral βLg appeared sufficiently long ago (>250 Mya) for its presence to be detectable now in almost all mammals. Its loss from the glires (rodents and lagomorphs) occurred when they and primates diverged about 80 Mya and a similar event may explain its absence in the Camelidae which diverged from the other artiodactyls (even-toed, hoofed mammals) about 40 Mya (Price et al., 2005; Wu et al., 2014). Although there appear to be exceptions as noted above, Gd appears to be restricted to primates which started diverging 60–70 Mya (Dawkins, 2004; Schiefner et al., 2015).

Having now put the occurrence of βLg and Gd in context, it is the purpose of this article to review their properties relevant to their physiological functions.

## β-Lactoglobulin

β-lactoglobulin is a major component of bovine whey with properties that affect processing in the food industry. The extensive literature describing its behavior under these non-physiological conditions can be accessed through Boland and Singh (2020) and references therein. There have also been thorough reviews of βLg over the years, beginning with Tilley (1960), covering the properties and structure of βLg, sometimes alone but also as part of wider reviews on milk proteins, all of which rather mirror the state of protein chemistry at the time. Sawyer (2003, 2013) and Edwards and Jameson (2020) are three of the more recent.

Early work on the nature of βLg showed that it contained a good distribution of essential, or indispensable, amino acids (see for example, Forsum and Hambraeus, 1974; Smithers, 2008) in consequence of which it has a clear nutritional role. This is hardly surprising as colostrum and milk are the sole food source in the first few days for the newborn (Levieux and Ollier, 1999; Levieux et al., 2002; Hambræus and Lönnerdal, 2003) and it seems reasonable to assume that the composition is optimized for each species (e.g., Beck et al., 2015). This nutritional role includes its being a ready source of bioactive peptides (Pihlanto-Leppala, 2000; Korhonen and Pihlanto, 2006; Hernández-Ledesma et al., 2008; Nielsen et al., 2017) that appear to be important in neonate development (Park and Nam, 2015; Dave et al., 2016). However, most of the recent nutritional studies discuss milk proteins in the context of human nutrition (e.g., Sánchez and Vázquez, 2017) and studies on milk derived bioactive peptides with reference to human well-being (e.g., Marcone et al., 2017). Bioactive peptides with antibacterial (Pellegrini et al., 2001; Pellegrini, 2003; Chaneton et al., 2011; Sedaghati et al., 2015), opioid (Chiba and Yoshikawa, 1986; Pihlanto-Leppala, 2000; Teschemacher, 2003) and antihypertensive (Dave et al., 2016) activities may well have such effects in the neonate animal for which they have evolved but do not appear to have been reported specifically, despite the growing interest in their use in animal nutrition (Hou et al., 2017). Similarly, while studies of satiety are of considerable interest in maintaining well-being (Kondrashina et al., 2020), studying such a topic in neonate animals is less straightforward although there are studies on peptide production from whey and βLg in pigs (Barbé et al., 2014), rats (Hernández-Ledesma et al., 2007) and, of course, humans (Boutrou et al., 2015).

On the above theme, are there correlations between βLg and production traits that are beneficial for the calf? As livestock are valuable, farmers presumably select for traits that enhance their profit (more milk; better meat; and healthier offspring). A healthy immune system and gut microbiome are obvious consequences of satisfactory colostrum and milk ingestion but so too are normal behavioral characteristics as has been examined in for example, cows (Krohn et al., 1999) or piglets (Prunier et al., 2020). The recent reports of the generation of animals in which the βLg gene has been switched off or knocked out (Lamas-Toranzo et al., 2017; Sun et al., 2018; Yuan et al., 2020), should provide clues to any possible function in the neonate other than nutrition.

Transfer of immunity from mother to offspring is species dependent (Larson, 1992; Langer, 2009; Pentsuk and van der Laan, 2009; Hurley and Theil, 2011; Butler et al., 2015). In some species, like human and rabbit, mothers pass significant amounts of immunoglobulin, mostly IgG, *in utero* before birth and the colostral antibody is mostly IgA. It has been shown that vaccinating pregnant women against tetanus, influenza and whooping cough (Lindsey et al., 2013) is generally beneficial for the infant but such immunization may interfere with the infant’s own immune response (Bergin et al., 2018; Orije et al., 2020). Other animals like horse, pig and cow transfer mostly IgG in the colostrum after birth and there is a final group that includes dog, cat and rodents that transfer immunoglobulins both *in utero* and via the colostrum (Larson, 1992). The neonate intestine in those species where transfer occurs after birth, is permeable to immunoglobulins for periods of a day to a few weeks (Staley and Bush, 1985; Sangild et al., 1999) to facilitate this uptake. βLg is resistant to low pH and to pepsin so that it is able to pass through the stomach more or less intact (Miranda and Pelissier, 1983; Guo et al., 1995; Almaas et al., 2006; Rahaman et al., 2017). In the intestinal tract, the pH rises and the protein becomes less stable and susceptible to enzymic hydrolysis (Guo et al., 1995). That βLg might in some way be related to antibody transfer was suggested by Jenness (1979) though without much conviction. There is little on the topic until Fleming et al. (2016) found a positive correlation between levels of IgG and βLg in herds of cows being classified as low, medium or high immune responders but more convincingly, Crowther et al. (2020) have shown that βLg associates fairly specifically with the immunoglobulin fraction of both cow and goat milk, their thesis being that such an association would protect the immunoglobulins during their passage through the stomach. However, this novel finding needs to be investigated further by identifying the exact nature of the interaction in ruminant and in other species. Such an interaction might be expected to be with the constant rather than the variable regions of the immunoglobulins. In this regard, the only structure of a βLg complex with the Fab fragment of a monoclonal IgE molecule raised against the milk protein is not relevant (Niemi et al., 2007).

Conversely, there is a large body of evidence that milk allergy, especially in infants, arises from the presence of βLg (Tsabouri et al., 2014; Linhart et al., 2019). Indeed, βLg is also known as Bos d5 allergen (Breiteneder and Chapman, 2014; Pomés et al., 2018), one of 12 cow allergens of which, Bos d2, is another lipocalin (Rouvinen et al., 1999). A recent report on βLg’s ability to promote proliferation of mouse hybridoma cells thereby enhancing an immune response (Tai et al., 2016), has not been shown in bovine cells but might be indicative of such a function in the immature calf intestine. Repeating such a study in a bovine mammary epithelial cell line (e.g., Huynh et al., 1991; German and Barash, 2002; Janjanam et al., 2013) would support this suggestion but the production of animals whose milk is without βLg (Lamas-Toranzo et al., 2017; Sun et al., 2018; Yuan et al., 2020) might better reflect the basis of their immunological well-being. Studies on the various epitopes identified on bovine βLg involve the use of antibodies raised in other species and consequently do not necessarily identify sites that are important in neonatal physiology (Williams et al., 1998; Clement et al., 2002; Cong and Li, 2012).

Although Davis and Dubos (1947) noted that βLg bound about half as much oleic acid as serum albumin, it was Groves et al. (1951) who showed that 2 mol/mol of sodium dodecyl sulfate not only bound but had a stabilizing effect on thermal denaturation. Since then, a large number of ligands for βLg has been identified and that number is still increasing (Sawyer, 2003; Tromelin and Guichard, 2006; Cherrier et al., 2013; Le Maux et al., 2014; Loch et al., 2015, 2018). To date, the only definitive ligand binding site is within the central calyx despite there being several experimental studies indicating that alternative sites may exist (Frapin et al., 1993; Dufour et al., 1994; Lange et al., 1998; Narayan and Berliner, 1998; Lübke et al., 2002; Yang et al., 2008, 2009; Edwards and Jameson, 2020). The crystal structures of many have been described (Qin et al., 1998; Wu et al., 1999; Kontopidis et al., 2002, 2004; Yang et al., 2008; Loch et al., 2012, 2013a,b, 2014; Rovoli et al., 2018) and some important NMR work has added to the description of the ligand binding site (Collini et al., 2003; Ragona et al., 2003, 2006; Konuma et al., 2007), in particular its pH dependence (Ragona et al., 2003). What is clear, however, is that the majority of molecules that bind are hydrophobic, or at least have significant hydrophobic moieties (Sawyer, 2013). This together with the similarity to other lipocalin transporters, most notably retinol-binding protein, has led to the speculation that βLg’s function is as a transporter (e.g., Sawyer, 2013; Edwards and Jameson, 2020). Further weight is given to this idea by the identification of specific βLg uptake in part of the intestine of the neonate calf (Papiz et al., 1986), a process lost in more mature intestine. There is evidence, however, that not every species has a βLg that can bind a ligand (Pérez et al., 1993). What might be the natural ligand? Fatty acids seem unlikely as they are more efficiently carried in fat globules. Vitamins A and D have been shown to bind and the amounts required are more in keeping with the 125 μM βLg present in cow’s milk but here too, hydrophobic vitamins are more likely found in the fat phase. Analysis of the ligands bound to βLg in milk showed only fatty acids (Pérez et al., 1989).

If transport is a function, then delivery implies some form of release mechanism as with RBP, or a receptor. Retinol is delivered by RBP/transthyretin to a surface receptor which internalizes the ligand only (Kawaguchi et al., 2007; Redondo et al., 2008). Papiz et al. (1986) reported the presence of specific βLg receptors in the neonate calf intestine, prompting speculation of the possible specific uptake of sparingly soluble ligands while Said et al. (1989) reported the βLg-enhanced uptake of retinol by suckling rats. Alternatively, the carrier plus cargo may be endocytosed. Reports of possible βLg receptors in rabbit ileum cells (Marcon-Genty et al., 1989), bovine germ cells (Mansouri et al., 1997), a CaCo-2 cell monolayer (Puyol et al., 1995) and a mouse hybridoma cell line (Palupi et al., 2000) have been followed by a description of the specific cellular uptake of βLg by the lipocalin-interacting membrane receptor (LIMR, Fluckinger et al., 2008). Although the LIMR used was human, there is a bovine receptor whose sequence is 59% identical (NCBI Reference Sequence: NP_001069254.2; Zimin et al., 2009). A more recent study of the human receptor, however, finds LIMR to be specific for human lipocalin-1 and nothing else (Hesselink and Findlay, 2013). There are receptors in the bovine intestine for various bioactive peptides generated by hydrolysis, but a specific receptor for βLg does not appear to have been reported since Papiz et al. (1986).

The pH dependent behavior of βLg was noted as early as by Pedersen, 1936 but it was the work of Tanford and coworkers which identified an anomalous carboxylate, known now to be Glu89, that was revealed by a conformational change at about pH 7, the “Tanford transition” (Tanford et al., 1959; Tanford and Taggart, 1961). Crystallographic (Qin et al., 1998; Vijayalakshmi et al., 2008; Labra-Núñez et al., 2021) and NMR (Uhrinova et al., 2000; Sakurai and Goto, 2006; Sakurai et al., 2009) structural work identified the conformational change as being the EF loop moving away from the entrance to the calyx thereby facilitating ligand binding (Figure 1B; Ragona et al., 2003; Konuma et al., 2007). The cow protein now has complete 3-dimensional structural data from pH 2 to 8 (Khan et al., 2018; Yeates and McPherson, 2019). Interestingly, the transition is shifted to significantly higher pH in porcine βLg (Ugolini et al., 2001) while the EF loop is also mobile in the protein structures available for sheep (Kontopidis et al., 2014; Loch et al., 2014), goat (Crowther et al., 2014; Loch et al., 2015), reindeer (Oksanen et al., 2006) and pig (Hoedemaeker et al., 2002). As Glu 89 is very well conserved among the βLg homologues, it may be that there is functional significance in this observed gating (Qin et al., 1998; Ragona et al., 2003; Konuma et al., 2007; Loch et al., 2019), mimicked by simulation (Bello and García-Hernández, 2014; Bello, 2020; Fenner et al., 2020; Labra-Núñez et al., 2021), once again being consistent with a transport function (e.g., Sawyer, 2013; Edwards and Jameson, 2020).

The ruminant βLgs are dimers at around neutral pH but become monomeric at low pH (Timasheff and Townend, 1961; Zimmerman et al., 1970; Joss and Ralston, 1996; Mercadante et al., 2012; Khan et al., 2018). The dimer interface involves the antiparallel arrangement of β-strand I as well as other interactions and crystal structures reported over a wide range of pH show that the interface is flexible (Vijayalakshmi et al., 2008; Crowther et al., 2016). Porcine βLg on the other hand is dimeric at low pH and monomeric around neutrality (Ugolini et al., 2001) with a completely different, domain-swapping dimerization (Hoedemaeker et al., 2002). The final species for which there is some structural information is equine βLg which is monomeric over a wide pH range (Kobayashi et al., 2000). A chimeric version, Gyuba βLg, with cow core and equine loops dimerises like the ruminant proteins (Ohtomo et al., 2011). When the horse I strand and AB loop were replaced by the cow amino acids, no dimer formed (Kobayashi et al., 2002). While the calyx opening is away from the dimer interface, structural and modeling studies of the ligand binding behavior show some dependency upon the quaternary structure (Bello et al., 2011, 2012; Domínguez-Ramírez et al., 2013; Gutiérrez-Magdaleno et al., 2013; Labra-Núñez et al., 2021). However, it is not clear whether the quaternary structure is important for any functional property of βLg, as it is for Gd.

Finally, is there evidence of the involvement of βLg in the mammary gland before or during lactation? Reinhardt and Lippolis (2006) showed that βLg was not present in the milk-fat globule membrane (MFGM) while Bianchi et al. (2009) showed its presence in milk-fat globules. A subsequent study of the MFGM proteins in engineered and cloned animals found no greater changes in expression levels of βLg between the engineered animals expressing human proteins than between the cloned control and normally bred animals (Sui et al., 2014). These studies, however, have little bearing on whether βLg is providing any specific function in the mammary gland. Both Ca^2+^ and Zn^2+^ bind to βLg and both ions are important mediators of metabolic function. Farrell and Thompson (1990) suggested such a role for calcium ions but the idea does not appear to have been revisited. The dissociation constant for Ca^2+^ is around 5 mM (Jeyarajah and Allen, 1994) which is tenfold higher than the concentration of βLg in milk. However, that for Zn^2+^ is about 5 μM (Tang and Skibsted, 2016) which makes an intracellular association with βLg possible. It is not clear that this is physiologically important either in mammary metabolism or as a means of ensuring the neonate has sufficient zinc (McCormick et al., 2014). Removal of βLg by genetic manipulation in cattle does not appear to cause any functional problem although there is a compensating increase in the amount of casein and α-lactalbumin (Jabed et al., 2012; Wei et al., 2018). However, in a similar study with goat, removal of βLg also led to a lowering of amounts of casein and lactalbumin (Zhou et al., 2017). Thus, while it is probably too soon to rule out any functional involvement of βLg in the mother, that cannot be said of its close relative, Gd.

## Glycodelin

The first reports of Gd appeared in the 1970s although, since the name tended to reflect the tissue from which the isolation had been prepared, the protein was referred to variously as progesterone-dependent or progesterone-associated endometrial (glyco)protein (PEP or PAEP, Joshi et al., 1980a, b), placental protein 14 (PP14, Bohn et al., 1982), placental α_2_-globulin (Petrunin et al., 1976), pregnancy-associated α_2_-globulin (α_2_-PEG, Bell et al., 1985a, b), chorionic or placental-specific α_2_-microglobulin (Petrunin et al., 1978; Tatarinov et al., 1980), or α_*2–uterine*_ protein (Sutcliffe et al., 1980). Bell and Bohn (1986) presented a discussion of this variability in nomenclature but the name glycodelin was not coined until Dell et al. (1995) to reflect the importance of glycosylation in the activity of the protein and to avoid using names apparently restricting its expression to specific tissues, and that is the name by which it will be referred to here. *PAEP* is used to refer to the gene (Kämäräinen et al., 1991; Van Cong et al., 1991; Uchida et al., 2013). However, as already noted *PAEP* is now also used to describe the βLg gene, rather than *LGB* or *BLG* (e.g., Elsik et al., 2016; Hunt et al., 2018; HGNC, 2020).

**FIGURE 3:**
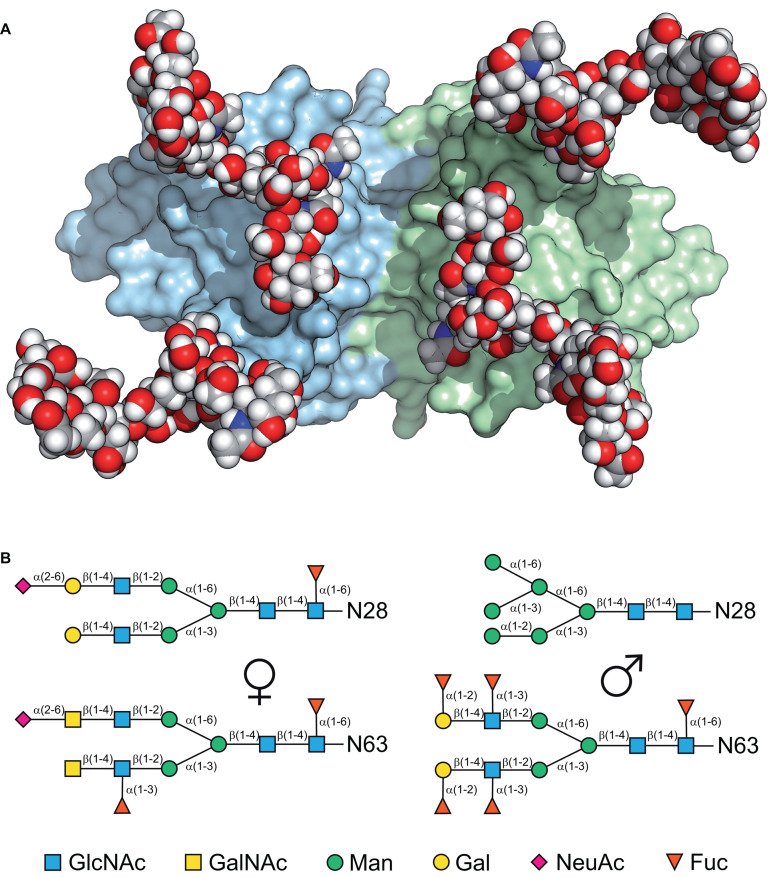
**(A)** A model of the glycosylated physiological dimer of Gd-A viewed down its two-fold axis, showing the two subunits as surface representations in pale green and blue with the branched sugar moieties on Asn28 and Asn63 shown as space-filling with the conventional elemental coloring. **(B)** Schematic representation of the typical major glycan structures found in Gd-A, a female amniotic fluid form on the left with that of Gd-S, the male seminal fluid form on the right (Dell et al., 1995; Morris et al., 1996). **(A,B)** Reproduced from Schiefner et al. (2015) with permission.

Glycodelin is implicated in the immunosuppression, angiogenesis and apoptosis activities associated with the first trimester of human pregnancy (Lee et al., 2016) as well as the fertilization and implantation processes (Seppälä et al., 2005, 2007; Lee et al., 2009). Its synthesis is therefore tightly controlled by progesterone, and possibly other factors like human chorionic gonadotrophin (hCG), relaxin and histone acetylation (Seppälä et al., 2009; Uchida et al., 2013). There are four distinct characterized isoforms of Gd all based upon the same polypeptide chain but differing in their glycosylation: Gd-A is found in amniotic fluid, in the secretory and decidualized endometrium (Seppälä et al., 2002; Koistinen et al., 2003) and in the serum of pregnant women (Bersinger et al., 2009), Gd-C is associated with the cumulus matrix (Chiu et al., 2007a), Gd-F occurs in follicular fluid and oviduct and Gd-S is expressed in seminal vesicles and found at high levels in seminal plasma (Yeung et al., 2006; Chiu et al., 2007b; Uchida et al., 2013). The differences in activity are dictated by the different oligosaccharides attached to Asn28, located in a loop at the end of β-strand A, and Asn63, located in the loop joining β-strands C and D (Schiefner et al., 2015). There are glycosylation differences not only between the tissues in which the Gd is found but also in the same tissue from different individuals (Koistinen et al., 1996, 2003). Figure 3 shows both the structure of the protein dimer with modeled sugars, and the distinct glycosylation patterns of Gd-A and Gd-S reflecting a distinction between female and male post-translational processing (Dell et al., 1995; Morris et al., 1996; Lapid and Sharon, 2006; Clark and Schust, 2013). Although there is a putative glycosylation site at Asn85, this is not modified, possibly because it is situated near the C-terminal end of β-strand E rather than in a loop at the end of the strand (Aubert et al., 1981; Moremen et al., 2012). Some of the immunomodulatory activity of Gd, however, appears to be associated with the protein moiety (Jayachandran et al., 2004; Ponnalagu and Karande, 2013; Dixit and Karande, 2020; Hansen et al., 2020) and it has been shown that Gd-A has lectin-like behavior in its interaction with T-cells (SundarRaj et al., 2009). However, much Gd binding involves its glycosylation (e.g., Lee et al., 2019; Dixit and Karande, 2020; Vijayan et al., 2020). Two of the Asn residues in Gd, those at 28 and 85, are Asp in βLg while it is the Ser65Glu change that disrupts the third N-linked glycosylation site (e.g., Sawyer, 2013). When these positions in βLg are engineered to those of Gd and the protein expressed in *Pichia pastoris*, glycosylation is observed but only at positions 28 and 63, that at 85 remaining unmodified as in Gd (Kalidas et al., 2001). It is not perhaps surprising to find that there is significant overlap in the epitope sequences in Gd and βLg as these tend to be on exposed sections of the polypeptide: angiogenic activity in Gd between 68 and 83 which includes the loop between strands D and E, and immunosuppressive activity between 57 and 65 at the other end of strand D (Ponnalagu and Karande, 2013). In βLg, these same regions have been identified as epitopes for human IgE and IgG (Cong and Li, 2012) which may indicate possible interaction sites in the calf. Results from Tai et al. (2016) show cellular proliferation via IgM but no epitope is identified, though lysine modification abolishes the effect.

The development of the human placenta in early pregnancy depends, *inter alia*, upon Gd-A, secreted by the endometrium in response to progesterone, and perhaps also hCG and relaxin, and which interacts with various cell types, especially the trophoblast and immune cells, modifying their behavior to allow implantation and ensure maternal tolerance of the growing embryo (Seppälä et al., 2002, 2007; Lee et al., 2011, 2016). It has also been found (Uchida et al., 2005, 2013) that Gd expression is regulated by histone acetylation/deacetylation such that overall control of expression appears to be both hormonal and epigenetic. Gd-A can also bind to the sperm surface through fucosyltransferase (Chiu et al., 2007b) inhibiting the sperm’s ability to penetrate the zona pellucida (Oehninger et al., 1995). Low levels of Gd-A are observed in both uterine flushings and the serum, during the fertilization window (e.g., Bersinger et al., 2009; Seppälä et al., 2009). Levels rise after the hormone burst at ovulation such that successful implantation only occurs in the presence of Gd-A (Kao et al., 2002), borne out by women with repeated implantation failure having low serum and endometrial levels (Bastu et al., 2015). The changes to the decidual leukocyte populations caused by Gd-A are also important for successful pregnancy (Erlebacher, 2013). T-helper type 1 cells are depleted along with B-lymphocytes while T-helper type 2 cells increase (Saito et al., 2010). Natural killer cells account for the majority of decidual leucocytes by the end of the first trimester but have a low cytotoxicity compared to those in blood (Erlebacher, 2013). During the preimplantation stage, monocytes migrate to the endometrium where they differentiate into decidual macrophages whence the interaction with Gd-A appears to maintain the survival of the fetus and placenta (Stout and Suttles, 2004; Lee et al., 2016; Vijayan et al., 2020). A decrease in the levels of Gd-A leads to an increase in interferon-γ causing a variety of problems from pre-eclampsia to fetal loss (Lee et al., 2016).

Gd-F is the isoform secreted into the follicular fluid, after synthesis in the granulosa cells (Tse et al., 2002; Chiu et al., 2003, 2007b). Through a high affinity site and a low affinity one (which also binds Gd-A), it binds to spermatozoa inhibiting both their interaction with the zona pellucida and the progesterone-induced acrosome reaction (Chiu et al., 2003; Yeung et al., 2009).

The oocyte-cumulus complex is released from the ovulatory follicle and transported, along with follicular fluid, through the oviduct after ovulation. Spermatozoa must pass through the follicular fluid and then negotiate the expanded cumulus complex, a sticky mass of cumulus cells and hyaluronic acid surrounding the zona pellucida and called the cumulus-oophorus, before they can bind to the zona pellucida and initiate fertilization. Gd-A and Gd-F both of which can bind to spermatozoa and prevent binding to the zona pellucida as noted, are present in the oviduct. However, it is a third form, Gd-C, generated in the cumulus cells by modification of the glycosylation of Gd-A and Gd-F that effectively removes their inhibition, allowing penetration and subsequent fertilization (Chiu et al., 2007a; Lee et al., 2009).

Gd-S is the male form of Gd found in large quantities in seminal plasma. It binds to spermatozoa inhibiting the loss of cholesterol which in turn would lead to premature capacitation before entry into the uterine cavity. Once in the uterine cavity, Gd-S is released, there is an efflux of cholesterol and capacitation occurs. Gd-F binds to prevent the acrosome reaction, the necessary prelude to zona penetration. Gd-S does not inhibit interaction between zona pellucida and spermatozoa and is not therefore contraceptive (Koistinen et al., 1996; Morris et al., 1996). There are two binding sites for Gd-S on the human spermatozoon which are distinct from those of the other isoforms (Chiu et al., 2005; Yeung et al., 2006). As noted above, the glycosylation is also distinct from the female forms of Gd in that it has no sialyl glycans but rather is fucose-rich (Morris et al., 1996).

The isoforms of Gd are therefore intimately associated with the human/primate reproductive process but in non-primate reproduction which must have arisen before the emergence of Gd (Puga Molina et al., 2018) there exist lipocalin alternatives, though not necessarily carrying out similar functions. For example, progesterone-dependent uterine lipocalins are found in the endometria of pig (RBP, Stallings-Mann et al., 1993; SAL1, Spinelli et al., 2002; Seo et al., 2011), mare (p19 or uterocalin, Crossett et al., 1996, 1998; Suire et al., 2001) and cow (RBP, Mullen et al., 2011) though none is closely related to either Gd or βLg. However, both p19 and SAL1 are glycosylated. In all of these animals, and ungulates in general, there is a significant delay before implantation during which the uterine secretions supply a wide range of molecules for nutrition, protection and development (Artus et al., 2020). RBP has also been identified in cat conceptus (Thatcher et al., 1991) and dog endometrium (Buhi et al., 1995). Finally, mouse oviduct and endometrium secrete lipocalin-2 which leads to sperm capacitation (Watanabe et al., 2014). Would it be more than coincidence that another lipocalin, βLg, with binding properties similar to these uterine ones is involved at a subsequent developmental stage?

Glycodelin is also implicated in other biological processes (Seppälä et al., 2007, 2009) most notably cancer (Richter et al., 2007; Kölbl et al., 2014; Cui et al., 2017) where its presence can indicate a better outcome in some ovarian and breast cancers (Mandelin et al., 2003; Koistinen et al., 2009; Hautala et al., 2011), supported by functional studies (e.g., Kämäräinen et al., 1997; Hautala et al., 2008). However, in other cancers, high Gd expression indicates a poorer prognosis, also supported by functional studies (e.g., non-small cell lung cancer, Schneider et al., 2015, 2018; malignant pleural mesothelioma, Schneider et al., 2016; melanoma, Ren et al., 2010). It has also been shown that the glycosylation pattern of Gd expressed in cancer cells is not the same as that in normal tissue expression (Hautala et al., 2020) though whether this has any functional relevance is unknown at present. The method used involved both antibodies and specific lectins, and may be of much wider application (Hautala et al., 2020). As the presence of Gd in tissues can be monitored by antibodies, both mono- and polyclonal and by mRNA there is scope for apparently conflicting observations. For example, it was found that the presence of Gd was indicative of a better prognosis in endometrial cancer whereas the presence of the immunosuppressive Gd-A indicated a poorer overall survival rate (Lenhard et al., 2013). In ovarian cancer too, the presence of Gd-A has a positive correlation with other markers that indicate a poorer outcome (Scholz et al., 2012; Ditsch et al., 2020). The presence of Gd can be detected by immunohistological staining of tissue biopsies and also in serum samples but is just one of many proposed neoplastic markers, the clinical significance of which, if any, as a cancer marker remains to be established.

The other clinical area in which Gd has been implicated arises from the fact that Gd levels are very low during the ovulatory phase of the human menstrual cycle, only to rise in tandem with progesterone levels. This has possible implications for contraception (Yeung et al., 2006) though it is not yet clear whether deliberate attempts to control Gd levels might provide an alternative approach to those methods currently in use. Studies to date have monitored Gd, Gd-A and progesterone levels throughout the cycle in the presence and absence of progestogen contraceptive treatment to identify the mechanism of action (e.g., Durand et al., 2010). Much of the research has been focused on the effects of the so-called “morning-after pill” emergency contraception (Durand et al., 2001, Durand et al., 2005, 2010; Vargas et al., 2012; Mozzanega et al., 2014) and opinion is split as to the mechanism of action of either of the two common drugs, levonorgestrel (LNG) or ulipristal acetate (UPA). Taken before ovulation, LNG raises the Gd concentration in serum and in uterine fluid before ovulation possibly inhibiting fertilization (Durand et al., 2010). Taken at or after ovulation, its effectiveness may involve sperm motility, capacitation or interaction with the zona pellucida though Gd-A alone was unable to mimic the effects (Chirinos et al., 2017). UPA on the other hand, has a decreasing effect as ovulation approaches but, since it blocks progesterone receptors, prevents implantation (Mozzanega et al., 2014). Mozzanega and Nardelli (2019) consider the mechanisms of LNG and UPA from the viewpoint of informed consent since there is significant ethical concern if the action is directed toward the conceptus (Kahlenborn et al., 2015; Peck et al., 2016).

## Conclusion

Glycodelin and β-lactoglobulin are two lipocalins with closely related sequences and 3-dimensional structures arising from homologous genes and both are involved in the reproductive process. The proposed (patho)physiological functions of Gd are several and well defined, at least in human cell models, and appear to be specifically dependent upon post-translational, N-linked glycosylation. βLg on the other hand appears much more widely distributed and is clearly important in the nutrition and health of the offspring, providing as it does, not only a balanced supply of amino acids, but also a series of peptides that have anti-oxidant and anti-bacterial properties which must help establish a good gut microbiome in the offspring. Several other activities have been associated with βLg, transport and, recently, immunoglobulin stabilization being perhaps the most likely. Now that animals can be produced which lack the protein, it should be possible to assess the nature of any problems that are manifest by its lack. However, today, the coin is still spinning!

## Data Availability Statement

The raw data supporting the conclusions of this article will be made available by the authors, without undue reservation.

## Author Contributions

The author confirms being the sole contributor of this work and has approved it for publication.

## Conflict of Interest

The author declares that the research was conducted in the absence of any commercial or financial relationships that could be construed as a potential conflict of interest.
